# Identification of Retinopathy of Prematurity Related miRNAs in Hyperoxia-Induced Neonatal Rats by Deep Sequencing

**DOI:** 10.3390/ijms16010840

**Published:** 2014-12-31

**Authors:** Ruibin Zhao, Lijuan Qian, Li Jiang

**Affiliations:** Department of Pediatrics, Zhongda Hospital, Southeast University, Nanjing 210096, China; E-Mails: tczhaoruibin@163.com (R.Z.); cqxbaby@163.com (L.Q.)

**Keywords:** retinopathy of prematurity, miRNAs, hyperoxia-induced rats

## Abstract

Retinopathy of prematurity (ROP) remains a major problem for many preterm infants. MicroRNAs (miRNAs) are a class of small noncoding RNAs that regulate gene expression at the posttranscriptional level and have been studied in many diseases. To understand the roles of miRNAs in ROP model rats, we constructed two small RNA libraries from the plasma of hyperoxia-induced rats and normal controls. Sequencing data revealed that 44 down-regulated microRNAs and 22 up-regulated microRNAs from the hyperoxia-induced rats were identified by deep sequencing technology. Some of the differentially expressed miRNAs were confirmed by quantitative reverse transcription-PCR (qRT-PCR). A total of 594 target genes of the differentially expressed microRNAs were identified using a bioinformatics approach. Functional annotation analysis indicated that a number of pathways might be involved in angiogenesis, cell proliferation and cell differentiation, which might be involved in the genesis and development of ROP. The elevated expression level of the vascular endothelial growth factor (VEGF) protein in the hyperoxia-induced neonatal rats was also confirmed by enzyme linked immunosorbent assay (ELISA). This study provides some insights into the molecular mechanisms that underlie ROP development, thereby aiding the diagnosis and treatment of this disease.

## 1. Introduction

The exposure of premature infants to hyperoxia or the relative hyperoxia of the nonuterine environment is associated with retinopathy. This retinopathy of prematurity (ROP) typically regresses but can lead to irreversible vision loss if progression from retinal neovascularization to cicatrization and retinal detachment occurs [1,2]. The most recent of several multicenter trials in which precise oxygen targeting was examined demonstrated an incidence of approximately 65% for ROP of any severity in a cohort of 24–28 week gestation preterm infants [3]. Great progress has been made over the last decade in identifying the biphasic progression of this disorder, which has been attributed to two major risk factors: low gestational age and use of supplemental oxygen. The first phase is initiated when the infant is born into a relatively hyperoxic environment, which causes vasoconstriction and arrested vessel growth that lead to retinal hypoxia. The second phase is induced by hypoxia, which causes elevated levels of growth factors, such as vascular endothelial growth factor (VEGF) and insulin-like growth factor-1 (IGF-1) with resultant vaso-proliferation, neovascularization, and potential retinal detachment with subsequent vision loss [4]. Thus, the relative hyperoxia followed by repetitive hypoxic episodes that is so common in preterm infants appears to play an important role in the development of ROP [5]. The understanding of this biphasic etiology of ROP has been enhanced by the development of rat pup models because the retinal vasculature of the newborn rat pup, which develops during the first two weeks of postnatal life, resembles that of a preterm infant [6]. However, additional efforts are needed to illustrate the molecular mechanism of ROP to develop more effective medical treatments.

MicroRNAs (miRNAs) are a class of small noncoding RNAs that regulate gene expression at the post-transcriptional level [7]. miRNA targeting is achieved by binding to complementary sites in the 3'-untranslated regions (3'-UTR) of messenger RNAs [8]. Gene expression regulation mediated by miRNAs is widespread in mammals; more than 1500 miRNA genes have been identified in the human genome (miRBase release 18.0 (http://microrna.sanger.ac.uk/)) [9,10], and it is estimated that one-third of genes are regulated by miRNAs [11]. miRNAs play key regulatory roles in cellular processes including apoptosis, proliferation and metastasis [12]. The aberrant expression of miRNAs has been reported to be involved in Burkitt’s lymphomas, B cell chronic lymphocytic leukemia (CLL) and many other solid cancer types, including breast, liver, ovarian, colorectal, prostate, and, recently, lung cancer [13,14]. However, only few reports have shown that miRNAs expressed in the eye are associated with physiologic and pathological processes and display tissue- and spatiotemporal-specific expression [15,16]. In mouse models of oxygen-induced retinopathy (OIR), miRNAs regulate retinal angiogenesis via the post-transcriptional modification of genes involved in the angiogenic response to hypoxia [17,18]. The miRNA internal reference gene expression in the rat retina was also reported by Tea *et al*. [19].

Recent findings suggest that circulating miRNAs can be used as biomarkers for tumor diagnosis and prognosis [20,21]. Genome-wide expression analyses of the miRNA from sera have been used to predict the survival of non-small-cell lung cancer (NSCLC) patients [22]. The latest studies reported by Zhang and Wu *et al.* also identified miRNAs in bronchopulmonary dysplasia from tissue-specific mouse models and preterm infants’ peripheral samples [23,24]. However, there are no reports of the potential relationship between serum/plasma miRNAs and retinopathy. In this study, we constructed two small RNA libraries from plasma samples from rat models of hypoxia and controls. Illumina technology was then used to sequence the two libraries and detect the genome-wide changes in miRNA expression. Fourteen miRNAs were discovered to be differentially expressed in the samples from the hypoxia model rats and the normal controls. The majority of the results were confirmed by quantitative reverse transcription-PCR (qRT-PCR), and enzyme-linked immunosorbent assay (ELISA) validations were also performed in several additional hyperoxia model rats. Functional annotation analysis of the target genes of these differentially expressed miRNAs indicated that these genes were overrepresented in the mitogen-activated protein kinases (MAPK), Wnt and nuclear factor-κB (NF-κB) signaling pathways, and in other pathways related to the retinopathy and VEGF. This study provides an improved understanding of the mechanism of ROP.

## 2. Results and Discussion

### 2.1. Analysis of Sequenced Small RNAs

To identify the differentially expressed miRNAs, we constructed two small RNA libraries from the plasma samples from the oxygen-induced rats and controls. A total of 3.27 million (controls) and 5.38 million (oxygen-induced rats: Hyperoxia) raw reads were generated from the two libraries. The results revealed that majority of reads had lengths of 18–25 nucleotides. The reads with lengths of 22 nt were the most abundant, followed the 23 and 21 nt-long reads, these lengths correspond to the average length of miRNAs (Supplementary Figure S1). Small RNAs of 20–24 nt in length accounted for 88.2% (controls) and 86.5% (Hyperoxia) of the total number of small RNA reads. After removing the contaminant reads, we obtained clean reads of 12–30 nt in length that accounted for 84.77% (controls) and 83.79% (Hyperoxia) of the total reads. The sequenced sRNAs were annotated as follows: miRNAs, mRNAs, repeats, other noncoding RNAs. As shown in Supplementary Figure S2, the proportion of known miRNAs increased from 11.70% in the samples from the controls to 14.35% in the samples from the hyperoxia, which implies that some of the miRNAs played important roles in the hyperoxia model-rats. In contrast, the proportion of unknown sRNAs increased from 41.06% in the controls to 42.48% in samples from the hyperoxia models, which suggests that unknown hyperoxia-related sRNAs remain to be identified.

### 2.2. Differential Expression Analysis of the miRNAs from the Hyperoxia Rats and Controls

**Figure 1:**
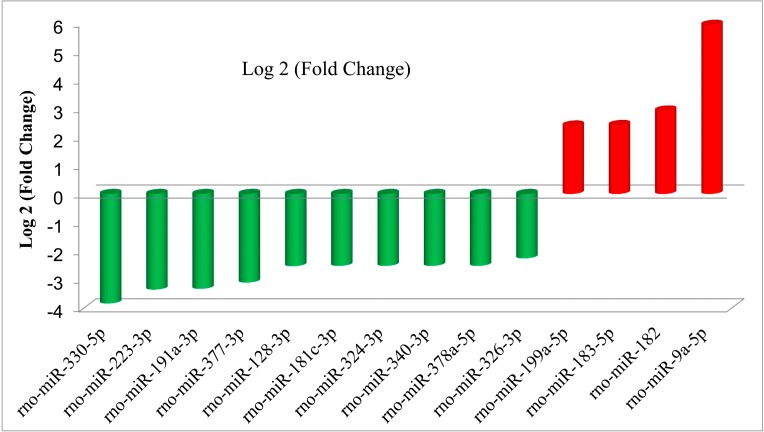
Significantly differentially expressed miRNAs in the plasma of the hyperoxia model rats. miRNAs that were up- and down-regulated in the hyperoxia model rats compared to the normal controls as defined by deep sequencing.

Based on high-throughput sequencing of the small RNAs, we performed differential expression analysis of the miRNAs from the two libraries from the hyperoxia rats and controls. We first removed the miRNAs with extremely low expression levels (normalization reads < 10). miRNAs were considered to be significantly up- or down-regulated if the Log 2 (case/control) were greater than 1 or less than −1, respectively, with a *p*-value below 0.05. Regarding the known miRNAs in the human genome, 66 (22 up-regulated and 44 down-regulated) known miRNAs were identified as differentially expressed between the hyperoxia rats and controls. As shown in Table 1 and Table 2, rno-miR-9a-5p was the miRNA that exhibited the greatest up-regulation in the plasma from the hyperoxia rats compared to the normal controls; these differences was an approximately a six-fold change. In contrast, rno-miR-330-5p, rno-miR-223-5p and rno-miR-191a-3p exhibited the greatest down-regulations, which were approximately four-fold changes. With stricter cut-off criteria (Log 2 (case/control) > 2), 14 miRNAs displayed considerable expression difference between the hyperoxia rats and the normal controls (all *p* < 0.001); four were up-regulated and 10 down-regulated (Figure 1). Supplementary Figure S3 showed the hierarchical clustering analysis of miRNAs expression in hyperoxia model rats compared to the control.

**Table 1:** Summary of down-regulated microRNAs (miRNAs).

Down-Regulated miRNAs	Log 2 (Fold-Change)
rno-miR-330-5p	−3.84522
rno-miR-223-3p	−3.35767
rno-miR-191a-3p	−3.33064
rno-miR-377-3p	−3.10825
rno-miR-128-3p	−2.535
rno-miR-181c-3p	−2.52329
rno-miR-324-3p	−2.52329
rno-miR-340-3p	−2.52329
rno-miR-378a-5p	−2.52329
rno-miR-326-3p	−2.26025
rno-miR-425-3p	−1.97075
rno-miR-191a-5p	−1.58496
rno-miR-122-5p	−1.52329
rno-miR-1306-5p	−1.52329
rno-miR-350	−1.52329
rno-miR-383-5p	−1.52329
rno-miR-483-3p	−1.52329
rno-miR-667-3p	−1.52329
rno-miR-708-5p	−1.52329
rno-miR-328a-3p	−1.50559
rno-miR-674-3p	−1.39776
rno-miR-423-3p	−1.3147
rno-miR-132-3p	−1.3009
rno-miR-342-3p	−1.28949
rno-miR-451-5p	−1.24595
rno-miR-345-3p	−1.23378
rno-miR-92a-3p	−1.17894
rno-miR-181d-5p	−1.14478
rno-miR-133a-3p	−1.1243
rno-miR-29a-3p	−1.10989
rno-miR-1843-5p	−1.10825
rno-miR-18a-5p	−1.10825
rno-miR-25-5p	−1.10825
rno-miR-324-5p	−1.10825
rno-miR-330-3p	−1.10825
rno-miR-3473	−1.10825
rno-miR-493-3p	−1.10825
rno-miR-872-3p	−1.10825
rno-miR-652-3p	−1.09265
rno-miR-505-3p	−1.0538
rno-miR-19b-3p	−1.04983
rno-miR-351-3p	−1.03786
rno-miR-126a-5p	−1.03563
rno-miR-17-5p	−1.02817

**Table 2:** Summary of up-regulated microRNAs (miRNAs).

Up-Regulated miRNAs	Log 2 (Fold-Change)
rno-miR-351-5p	1.036533
rno-miR-200c-3p	1.049601
rno-miR-219a-2-3p	1.061673
rno-miR-92b-3p	1.061673
rno-miR-200a-3p	1.077104
rno-miR-127-3p	1.107432
rno-miR-181a-2-3p	1.139676
rno-let-7e-5p	1.177151
rno-miR-433-3p	1.177151
rno-miR-337-5p	1.213677
rno-miR-494-3p	1.35118
rno-miR-200b-3p	1.424244
rno-miR-204-5p	1.512335
rno-miR-195-5p	1.535605
rno-miR-125b-5p	1.552595
rno-miR-3068-3p	1.564174
rno-miR-375-3p	1.747109
rno-miR-429	1.837458
rno-miR-199a-5p	2.406502
rno-miR-183-5p	2.424824
rno-miR-182	2.933923
rno-miR-9a-5p	5.936143

**Figure 2:**
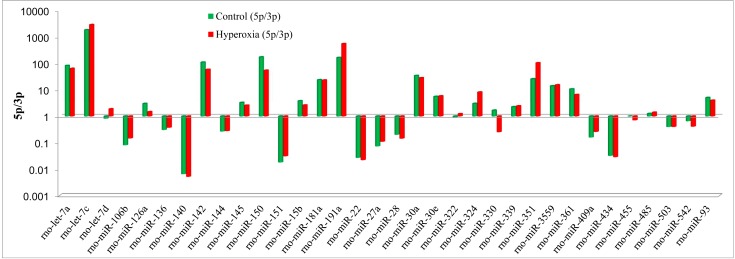
The 5p/3p ratios of the differential miRNAs exhibited significant differences between the hyperoxia model rats and the controls.

Interestingly, the ratio of 5p to 3p miRNA counts also exhibited significant differences in some of these differentially expressed miRNAs between the hyperoxia rats and the controls (Figure 2), which suggests that the ratios of 5p to 3p of certain miRNAs were involved and played important roles in the oxygen-induced model rats.

### 2.3. qPCR Validation of the Differential Expressions of the miRNAs

**Figure 3:**
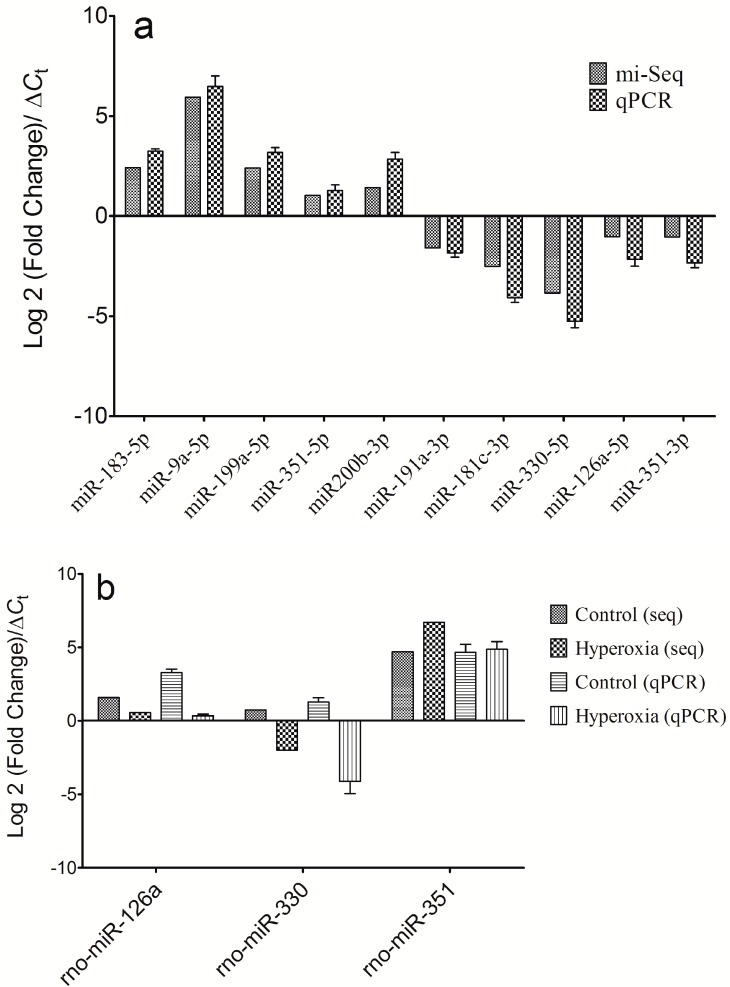
Comparison of the high-throughput sequencing and quantitative real-time PCR results. (**a**) The differentially expressed miRNAs, including rno-miR-183-5p, rno-miR-9a-5p, rno-miR-199a-5p, rno-miR-351-5p, rno-miR-200b-3p, rno-miR-191a-3p, rno-miR-181c-3p, rno-miR-330-5p rno-miR-126a-5p and rno-miR-351-3p were used to perform expression analyses by qPCR. Comparisons of the high-throughput sequencing and qRT-PCR results indicated that the two plarforms exhibited good correlation; and (**b**) The 5p/3p ratio validation results for miR-126a, miR-330 and miR-351 by qPCR also indicated good correlation between the two platforms.

To confirm the deep sequencing results, we used qRT-PCR to assess the expressions of 10 of the miRNAs (miR-183-5p, miR-9a-5p, miR-199a-5p, miR-351-5p, miR200b-3p, miR-191a-3p, miR-181c-3p, miR-330-5p, miR-126a-5p and miR-351-3p) in the 12-pair plasma samples from the hyperoxia rats and controls. Among these miRNAs, the 5p/3p ratios of miR-351, miR-330 and miR-126a were also measured for validation. These 10 selected miRNAs covered both highly expressed miRNAs and lowly expressed miRNAs that exhibited dysregulations in the hyperoxia rats compared to the controls. The expression levels of these 10 miRNAs were calculated using the comparative *C*_t_ method in which the *C*_t_ value of each miRNA is normalized to U6 snRNA using the comparative *C*_t_ (Δ*C*_t_). All 10 of the miRNAs were expressed at significantly different levels in the plasma samples from the hyperoxia rats and controls. As shown in Figure 3, the qPCR results demonstrated a very good correspondence between the two analysis methods.

### 2.4. miRNA Target Prediction and Functional Annotation

We used the online software TargetScan (http://www.targetscan.org/) with the default parameters combined with PicTar (http://pictar.bio.nyu.edu/) to predict the target genes of the differentially expressed miRNAs. A total of 594 target genes were identified for the known miRNAs. To describe the network of miRNAs and target genes involved in the hyperoxia rats, we constructed a regulatory network diagram. As shown in Supplementary Figure S4, a total of 603 nodes and 621 edges were included in this network.

**Figure 4:**
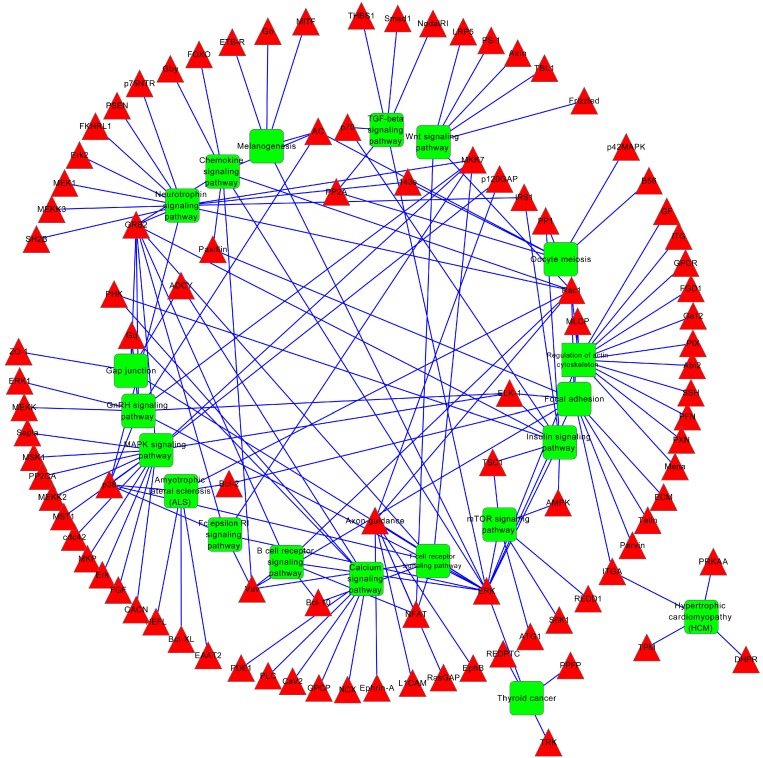
The predicted target genes involve the KEGG pathway-mediated regulatory network in the hyperoxia model rats. The red nodes represent the target genes of the differentially expressed miRNAs, and the green nodes represent the involved KEGG pathway.

To gain insights into the biological implications of the differentially expressed miRNAs, we assessed the miRNA target genes within the regulatory network for enrichment in Gene Ontology (GO) categories. The genes that exhibited nominal significances of *p* < 0.01 were selected and tested against the background set of all genes with GO annotations. We found some GO terms that were significantly enriched (FDR < 0.01) for these miRNA target genes, among which were GO processes that were related to their biological functions. The three GO classifications (*i.e*., molecular function, biological process and cellular component) were evaluated by level, but only the significant terms of the biological processes are listed in Table 3. From this table, it can be observed that most of the significant GO terms were related to development and cell morphogenesis, migration and motility. There were also a number of significantly enriched GO categories, including regulation of gene expression, regulation of cellular biosynthetic processes, and regulation of biosynthetic processes. Pathway analysis by integrating TargetScan, PicTar, and DIANA created a rank-ordered list of the 20 pathways with the most significant gene-enrichment (Table 4 and Figure 4) in which the differentially expressed miRNAs might be involved. Importantly, the top canonical pathways of the targets of the differentially expressed miRNAs included pathways related to cancer and endocytosis, protein processing in neurotrophin signaling pathway, chemokine signaling pathway, mitogen-activated protein kinases (MAPK) signaling pathway and TGF-β signaling pathway.

**Table 3:** The gene ontology (GO) terms predicted targets of the differentially expressed miRNAs.

GO Term	Gene Count	*p*-Value	FDR (Benjamini Hochberg) *
Embryonic development ending in birth or egg hatching	37	7.10 × 10^−10^	8.80 × 10^−7^
Chordate embryonic development	37	4.90 × 10^−10^	1.20 × 10^−6^
Intracellular signaling cascade	60	1.40 × 10^−8^	1.20 × 10^−5^
Cell migration	27	5.40 × 10^−8^	3.40 × 10^−5^
Cell motion	34	2.60 × 10^−7^	1.30 × 10^−4^
In utero embryonic development	24	4.40 × 10^−7^	1.80 × 10^−4^
Embryonic morphogenesis	28	1.20 × 10^−6^	4.10 × 10^−4^
Cell morphogenesis	28	3.30 × 10^−6^	1.00 × 10^−3^
Cell motility	27	4.80 × 10^−6^	1.30 × 10^−3^
Localization of cell	27	4.80 × 10^−6^	1.30 × 10^−3^
Positive regulation of gene expression	37	6.10 × 10^−6^	1.50 × 10^−3^
Embryonic organ development	21	6.70 × 10^−6^	1.50 × 10^−3^
Positive regulation of nucleobase, nucleoside, nucleotide and nucleic acid metabolic process	39	1.10 × 10^−5^	1.90 × 10^−3^
Cell projection organization	28	9.90 × 10^−6^	2.00 × 10^−3^
Forebrain development	18	1.00 × 10^−5^	2.00 × 10^−3^
Positive regulation of cellular biosynthetic process	41	1.50 × 10^−5^	2.40 × 10^−3^
Positive regulation of transcription	35	2.20 × 10^−5^	2.90 × 10^−3^
Appendage morphogenesis	13	2.30 × 10^−5^	2.90 × 10^−3^
Limb morphogenesis	13	2.30 × 10^−5^	2.90 × 10^−3^
Positive regulation of biosynthetic process	41	2.20 × 10^−5^	3.00 × 10^−3^

* FDR: False Discovery Rate.

**Table 4:** The Kyoto Encyclopedia of Genes and Genomes (KEGG) terms of the predicted targets of the differentially expressed miRNAs.

KEGG Term	Gene Count	*p*-Value	FDR (Benjamini Hochberg) *
Neurotrophin signaling pathway	17	1.00 × 10^−6^	1.20 × 10^−4^
Regulation of actin cytoskeleton	16	1.60 × 10^−3^	6.10 × 10^−2^
MAPK signaling pathway	19	1.10 × 10^−3^	6.20 × 10^−2^
Focal adhesion	14	6.10 × 10^−3^	1.60 × 10^−1^
GnRH signaling pathway	9	7.50 × 10^−3^	1.60 × 10^−1^
mTOR signaling pathway	6	2.20 × 10^−2^	2.30 × 10^−1^
Melanogenesis	8	2.10 × 10^−2^	2.40 × 10^−1^
Chemokine signaling pathway	12	1.50 × 10^−2^	2.50 × 10^−1^
Oocyte meiosis	9	1.90 × 10^−2^	2.50 × 10^−1^
Insulin signaling pathway	10	1.90 × 10^−2^	2.70 × 10^−1^
Amyotrophic lateral sclerosis (ALS)	6	3.30 × 10^−2^	2.80 × 10^−1^
Wnt signaling pathway	10	3.20 × 10^−2^	2.90 × 10^−1^
ErbB signaling pathway	7	4.40 × 10^−2^	3.10 × 10^−1^
TGF-beta signaling pathway	7	4.60 × 10^−2^	3.10 × 10^−1^
Maturity onset diabetes of the young	4	4.30 × 10^−2^	3.30 × 10^−1^
Vascular smooth muscle contraction	8	5.60 × 10^−2^	3.30 × 10^−1^
Dilated cardiomyopathy	7	5.50 × 10^−2^	3.40 × 10^−1^
Thyroid cancer	4	6.20 × 10^−2^	3.40 × 10^−1^
Natural killer cell mediated cytotoxicity	7	8.30 × 10^−2^	3.80 × 10^−1^
B cell receptor signaling pathway	6	7.70 × 10^−2^	3.90 × 10^−1^

* FDR: False Discovery Rate.

### 2.5. VEGF Level and Hyperoxia-Related miRNAs

**Figure 5:**
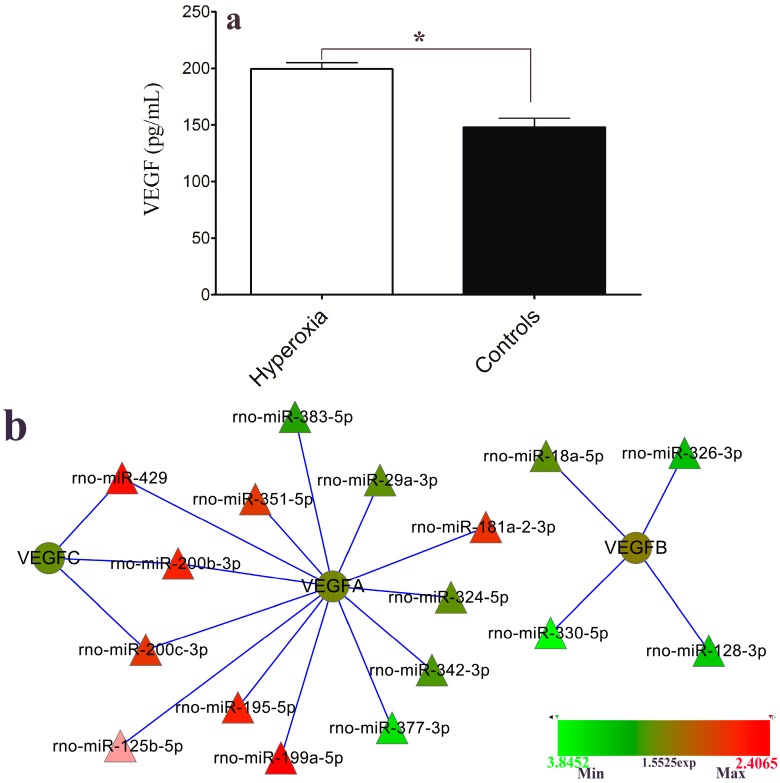
(**a**) The level of vascular endothelial growth factor (VEGF) protein secretion into the plasma of the hyperoxia model rats and controls were evaluated with enzyme-linked immunosorbent assay (ELISA) (mean ± SEM; * *p* < 0.01); and (**b**) Network interactions of the dysregulated miRNAs and the target gene *VEGF*.

VEGF was listed among the target genes of the differential miRNAs that were differentially expressed in the hyperoxia model rats in this study. The level of VEGF secretion into the plasma was evaluated with an enzyme-linked immunosorbent assay (ELISA). As shown in Figure 5a, the VEGF concentration in the hyperoxia group was significantly increased compared to that of the control group (*p* < 0.01). The interaction network of VEGF and its related miRNAs indicated that differential expressions were also demonstrated in this study. As shown in Figure 5b, VEGFA were targeted by the greatest number of the differential miRNAs, among which miR-429, miR-200b and miR-200c also interacted with VEGFC; in contrast, VEGFB was targeted only by miR-18a, miR-326, miR-330 and miR-128. The different colors of the miRNAs indicate dysregulation in the hyperoxia model rats; red indicates up-regulation, and green denotes down-regulation.

In the present study, we identified differentially expressed miRNAs in the plasma of hyperoxia model rats relative to controls with deep sequencing. Some of these significantly differentially expressed miRNAs were confirmed by qRT-PCR, which showed that the dysregulation of these miRNAs might have been caused by the condition of hyperoxia. Some of the miRNAs were also defined according to different 5p to 3p ratios between the hyperoxia and control rats, which implies that miRNA 5p/3p ratio differences regulate gene expression though interactions and might be associated with the development and progression of oxygen induced-ROP. Further in-depth studies of the molecular mechanisms underlying the development of ROP due to hyperoxia are warranted.

Further bioinformatics analyses could help us to investigate the roles of the deregulated miRNAs in this hyperoxia-induced rat model of ROP. Gene Ontology and KEGG pathway enrichment analyses were used to interpret the biological functions of the miRNA targets. GO analysis of the miRNA targets indicated that the miRNAs played important roles in embryonic development, cell morphogenesis, cell migration, regulation of gene expression, cell proliferation and regulation of biosynthetic processes. Among the predicted targets of these hyperoxia specific miRNAs, numerous vital genes are believed to participate in several important signaling pathways including those related to neurotrophin, MAPK, TGF-β, Wnt, insulin, ErbB and vascular smooth muscle contraction. Dysregulation of these miRNAs might function to induce angiogenesis and thereby contribute to the initiation and progression of ROP. The modulation of miRNA expression has great potential for ROP therapy. Therefore, our prediction that the differentially expressed miRNAs have the capability to target multiple components in these critical pathways makes them promising molecular targets for the treatment of ROP.

Our previous study showed that expression levels of VEGF mRNA and protein are significantly altered in metabolic acidosis-induced ROP model rats. In the present study, VEGF was also predicted to be a gene targeted by the specific hyperoxia-induced miRNAs, which was confirmed by ELISA. These analysis results revealed that the expression level of the VEGF protein was significantly higher in the hyperoxia-induced model rats. The interaction network analysis results revealed that VEGF-B was targeted by four down-regulated miRNAs and that VEGF-C was targeted by three up-regulated miRNAs, while VEGF-A was targeted by both up- and down-regulated miRNAs (Figure 5b). These findings suggest that the expression level of the VEGF protein can be dysregulated by miRNAs in different induction conditions. Related reports by Shen and Bai *et al*. revealed that alteration of miRNAs such as miR-126, miR-451, miR-199a, *et al*., contributed to the retinal neovascularization, which is consistent with our results [17,18].

## 3. Experimental Section

### 3.1. Experimental Animals and Treatment

#### 3.1.1. Experimental Animals

The Sprague-Dawley (SD) rats used in this study were purchased from the animal center of Nanjing Medical University. The rats were allowed unlimited access to rat chow and water and were exposed to a 12 h:12 h light-dark cycle. The temperature was maintained at 24 °C in a humidified atmosphere with daylight illumination. All experiments were approved by the Experimental Animal Ethics Committee of Zhongda Hospital, Southeast University.

#### 3.1.2. Exposure of Neonatal Rats to Cyclic Hyperoxia

In this study, OIR in the model rat was obtained according to the previous studies [25,26,27,28]. Within 12 h of birth, we put the mother rats and their offspring in a humidified chamber, which the neonatal rat is exposure to oxygen with control. The rats were exposed to 80% oxygen in air for 24 h and then 21% oxygen in air for 24 h, with this alternating 24 h cycles to 14 days after birth. Room air-exposed and age-matched neonatal rats were used as controls.

### 3.2. Plasma Separation and RNA Extraction

After the neonatal rats were exposed to hyperoxia, orbital blood samples from the OIR rats and the controls were treated and then mixed equally into two specimens respectively for further analyses. The model rats was in early stage of neovascularization phase when sampling. Briefly, 2 mL of blood was collected from each of the 12 hyperoxia rats and matched controls; the plasma samples were separated according methods described in previous studies [29]; and then pooled equally. Total RNA including miRNA was isolated from the plasma samples from the rat model of OIR and the controls using a miRNeasy Serum/Plasma Kit (Qiagen, Valencia, CA, USA) following the manufacturer’s instructions. The concentration of the RNA fraction was quantified using a Qubit^®^ RNA HS Assay Kit by Qubit^®^ 2.0 Fluorometer (Life Technologies, Grand Island, NY, USA), according to the manufacturer’s protocol. The quality of the obtained miRNA was measured with NanoDrop ND-1000 (NanoDrop, Wilmington, DE, USA).

### 3.3. Deep Sequencing

Small RNA libraries were constructed according to the Illumina^®^ TruSeq^®^ Small RNA Sample Preparation protocol (Illumina, San Diego, CA, USA) with some modifications as described in previous reports [30,31,32]. 3'-Adaptors and 5'-adaptors were ligated to the miRNA samples using T4 RNA ligase and were subsequently reverse-transcribed into cDNA with Superscript II Reverse Transcriptase and a size-selection performed with polyacrylamide gel electrophoresis was used to purify the library. A 6-nt index sequences was introduced during the PCR amplification. These two prepared libraries and the other indexed samples were mixed in a pool at the same concentration before the cluster generation. Library and cluster preparation was performed according to the standard Illumina protocol. We applied Illumina HiSeq 2500 to sequence the RNA libraries.

CAP-miRSeq was employed for data analysis according to the user guide with some modifications [33]. The mean length of the Illumina sequence reads was 41 nucleotides, which was greater than the mean size of the microRNAs (19–25 nucleotides). Because the reads contained part of the 3'-adaptor at the end of the sequences, we used Novoalign (version 2.08.01, Novocraft 2010; www.novocraft.com) to cut all reads at the 3'-end to remove the adapter sequences. After adaptor trimming, reads less than 12 bases were discarded. Next, the trimmed reads were input into miRDeep2 [34] to quantify the known miRNAs against the miRBase and to predict novel miRNAs. The expression values of miRNAs were selected for the differential expression analysis using the tool edgeR from Bioconductor as described previously [35].

### 3.4. qPCR Confirmation

qRT-PCR was performed to validate the expression levels of the analyzed miRNAs by sequencing. Reverse transcription was performed in a 20 µL reaction containing 2 µL of the ligation product, 1 µL of 5 µM RT primer, 1 µL of 10 mM dNTP, 4 µL of 5× PrimeScript Buffer, and 200 units of PrimeScript RTase (TaKaRa, Dalian, China). The reaction was incubated at 42 °C for 60 min, and then terminated by heating at 85 °C for 5 min. A no-template control (NTC) and no-reverse transcriptase control (−RT) were included in all RT reactions. The quantitative PCR amplification was performed using the SYBR Green I^®^ Roche, Basel, Switzerland) Quantitative Real-Time PCR (qPCR) Assay with individual specific primers according to the methods reported in previous study [36]. A dissociation curve was constructed for each reaction to verify the effectiveness. All reactions were run in triplicate, and the average threshold cycle and SD values were calculated. U6 snRNA was used as an internal reference control [19].

### 3.5. Prediction and Enrichment Analyses of the Target Genes

The target genes of the differentially expressed miRNAs were predicted using TargetScan software [11]. Gene Ontology (GO) [37] enrichment analyses of the target genes were performed to investigate the functional distribution of miRNAs specific to the hypoxia model rats. Additionally, KEGG pathway analyses of the target genes were also performed as previously described [38,39].

### 3.6. ELISA

Plasma samples from an additional five oxygen-induced model rats and three controls were used to measure VEGF content according to the method described in previous studies by Nanjing Realgene Biotech Co., Ltd. (Realgene, Nanjing, China) [40,41].

### 3.7. Statistics

For the qRT-PCR data, the relative expression levels of each target miRNA (Log 2 relative level) were calculated according to the difference in CT values between the target miRNAs and U6 snRNA (Δ*C*_t_). The statistical analyses were performed using SPSS software version 16.0 (SPSS Inc., Chicago, IL, USA) and GraphPad Prism 5 (GraphPad Software Inc., La Jolla, CA, USA). *p*-values < 0.05 were considered to indicate statistically significant differences (Student’s *t*-test).

## 4. Conclusions

In summary, this study described an initial use of deep sequencing to comprehensively profile the miRNAs in the plasma of hyperoxia-induced rats. Our comprehensive survey of the differentially expressed miRNAs not only confirmed some existing findings but also revealed dysregulated miRNAs that had not been previously been identified in studies with ROP models and thereby provided new signatures of ROP. The results presented in this study illustrate some of the underlying biological processes that might be involved in hyperoxia induced ROP.

## Supplementary Materials

Supplementary figures can be found at http://www.mdpi.com/1422-0067/16/01/0840/s1.
